# Macrophage-Osteoclast Associations: Origin, Polarization, and Subgroups

**DOI:** 10.3389/fimmu.2021.778078

**Published:** 2021-12-01

**Authors:** Yang Sun, Jiangbi Li, Xiaoping Xie, Feng Gu, Zhenjiang Sui, Ke Zhang, Tiecheng Yu

**Affiliations:** Department of Orthopedics, The First Hospital of Jilin University, Changchun, China

**Keywords:** macrophages, osteoclasts, associations, origin, polarization, cytokines, subgroups

## Abstract

Cellular associations in the bone microenvironment are involved in modulating the balance between bone remodeling and resorption, which is necessary for maintaining a normal bone morphology. Macrophages and osteoclasts are both vital components of the bone marrow. Macrophages can interact with osteoclasts and regulate bone metabolism by secreting a variety of cytokines, which make a significant contribution to the associations. Although, recent studies have fully explored either macrophages or osteoclasts, indicating the significance of these two types of cells. However, it is of high importance to report the latest discoveries on the relationships between these two myeloid-derived cells in the field of osteoimmunology. Therefore, this paper reviews this topic from three novel aspects of the origin, polarization, and subgroups based on the previous work, to provide a reference for future research and treatment of bone-related diseases.

## Introduction

The skeleton is a complex organ that facilitates locomotion, retains blood calcium concentration, provides stable support to soft tissues, and is a site for adult hematopoiesis. Continued bone remodeling is necessary to maintain these crucial functions by preventing the accumulation of bone injuries, and sustaining both calcium homeostasis and bone strength (1, 2). The remodeling of bone is a tightly-coupled process that involves osteoclasts and osteoblasts. Among them, osteoclasts are multinucleated cells that develop from the fusion of osteoclast precursors (OCPs) through the activation of macrophage colony‐stimulating factor (M‐CSF) and receptor activator of NF‐kB ligand (RANKL), which can secret H^+^, Cl^-^, cathepsin K (CtsK), and matrix metalloproteinases (MMPs) (3–5). Importantly, osteoclasts are the only bone-resorbing cells in the human body, and are crucial for remodeling of the skeletal system.

On the other hand, macrophages are phagocytes from the mononuclear myeloid lineage. They are known for their protective roles in eliminating pathogens and recruitment of other immune cells from the peripheral circulation to the sites of infection and inflammation. These cells have evolved; emphasizing their distinct and critical significance in almost all tissues. Moreover, most tissues and organs host a resident group of macrophages that have adapted to the local conditions and are able to perform crucial tissue-specific functions in order to maintain homeostasis (6). For instance, osteal macrophages, which are the resident tissue macrophages in bones, perform various functions in the bone microenvironment. Besides, macrophages can change their functional and phenotypic properties in response to signals released from the immediate environment (7). Based on their activation states, macrophages can be categorized into M1 and M2 subtypes (8). In fact, M1- and M2-like macrophages share several features with T helper (Th) 1/Th2 cells (9). It has been reported that M1 macrophages are antimicrobial and proinflammatory, whereas M2 macrophages are anti-inflammatory (10, 11). Various physiological processes, including skeletal homeostasis, depend on the balance between M1 and M2 macrophages.

In the field of osteoimmunity, immune and skeletal systems share numerous biological factors, such as chemokines, cytokines, hormones, etc. In addition, the interactions between osteoclasts and macrophages exert an indispensable influence in this area. Despite the well-established wealth of knowledge on macrophage genesis, activity, and polarization, the relationships between macrophages and osteoclasts in osteoimmunity still need further elucidation. In this article, we primarily focused on the effect of macrophage polarization on osteoclasts. We found that various cytokines, such as interleukins (ILs), chemokines, and tumor necrosis factors (TNFs) produced during the polarization process are intimately associate with he differentiation, activity, and survival of osteoclasts. Furthermore, we complementarily described the monocyte/macrophage origin of osteoclasts and the relationships between macrophage subpopulations and osteoclasts. In general, we summarized the current knowledge on macrophage-osteoclast interactions from diverse perspectives to provide references for future studies.

## The Monocyte/Macrophage Origin of Osteoclasts

Various theories have been propounded to account for the origin of osteoclasts since they were discovered in 1873 (12). However, Walker’s pioneering experiments in the 1970s confirmed their hematopoietic origin, as transfusions of spleen and myeloid cells from wild-type mice reversed bone resorption in osteosclerotic and osteopetrotic mice lacking osteoclasts. This demonstrated that hematopoietic organs are capable of generating cells that cause hard tissues to resorb (12, 13). In 1986, Scheven and coworkers were the first to show that osteoclasts can be produced from a subgroup of cells that are rich in hemopoietic progenitors (14). In 1990, the monocyte/macrophage origin of osteoclast was later certified by Udagawa and partners after they proved that hemopoietic stem cells differentiate into the monocyte-macrophages through the activation of M-CSF (15). Subsequent studies further confirmed that osteoclasts can be formed from monocyte-macrophage precursor cells as well as from mature macrophages in tissues (15).

Furthermore, bones and bone marrow contain three unique macrophage populations, namely: osteoclasts and bone marrow macrophages (erythroid island macrophages), hematopoietic stem cell macrophages, and a newly-discovered group of macrophages called osteal macrophages or “osteomacs” (16). This also suggests that osteoclasts and macrophages may have a similar origin. Studies have shown that cells from the mononuclear/macrophage system, which contain hematopoietic marrow cells, blood monocytes, and peritoneal macrophages, possess the ability to develop into bone-resorbing osteoclasts; thus, squarely classifying osteoclastic groups within these series of cells (17–20). In fact, osteoclast and macrophage are the two differentiation products from myeloid precursors that compete with each other (21). In summary, osteoclasts are generated by a series of processes that begins with the commitment of hematopoietic stem cells (HSCs) to the mononuclear/phagocyte series (22), followed by the proliferation of pre-osteoclasts, and final maturation into osteoclasts, which possess bone-resorbing capacity (23).

**Figure 1:**
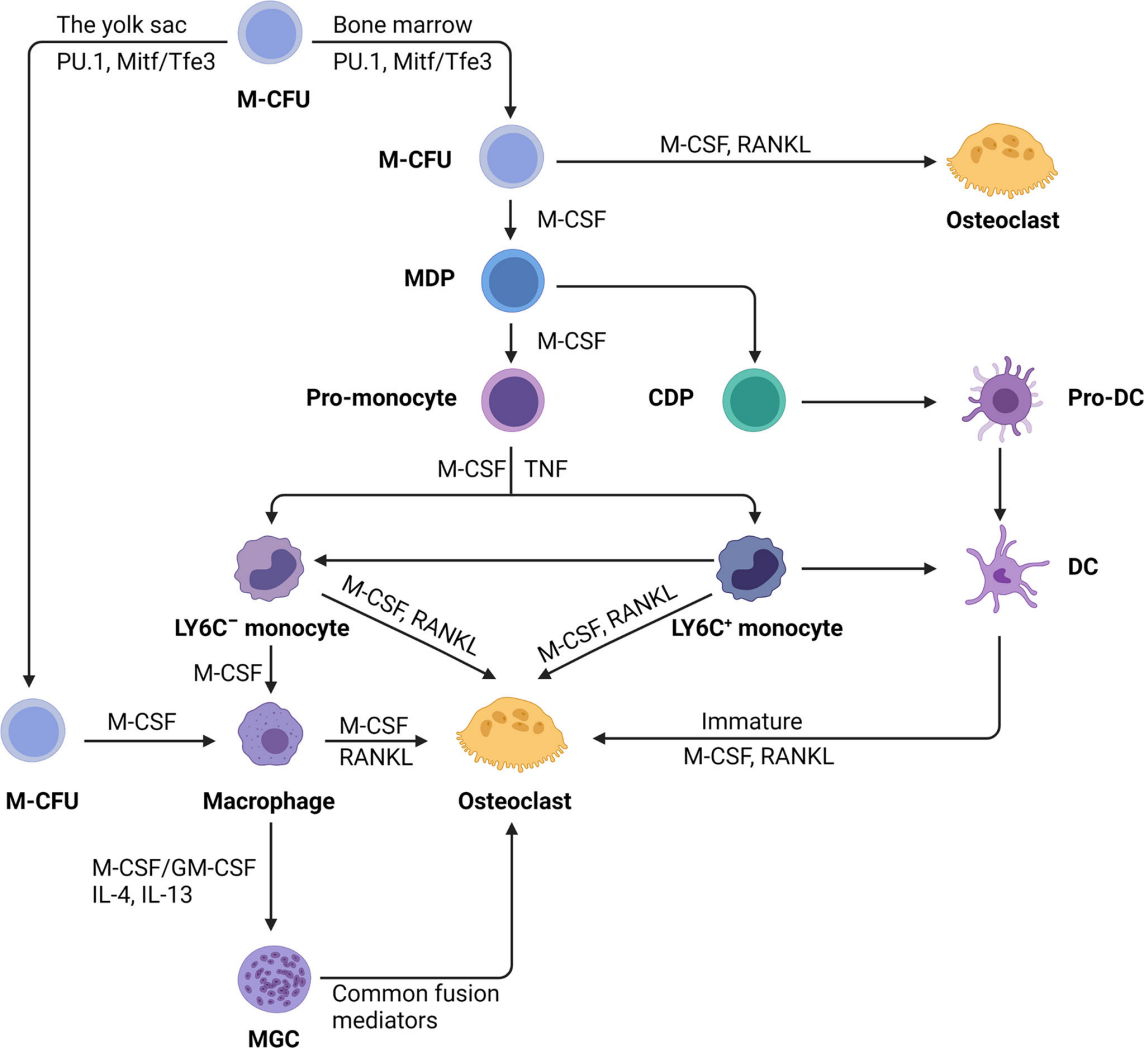
The monocyte/macrophage origin of osteoclasts. Hematopoietic stem cells myeloid colony-forming units (M-CFU) from bone marrow or yolk sac is the main site of myeloid cell production. Recent researches have shown that tissue-resident macrophages initially arise from myeloid stem cells (M-CFU) in the yolk sac of the developing embryo. In M-CFU, early expression of PU.1 and Mitf induces the emergence of M-CSFR (the receptor for M-CSF). Subsequently, in combination with macrophage colony‐stimulating factor (M-CSF) and receptor activator of NF‐κB ligand (RANKL), M-CFU can differentiate directly into osteoclasts. But in the separate action of M-CSF, M-CFU can differentiate into M-CSF-dependent macrophage and dendritic cell progenitor (MDP). MDP can differentiate into both dendritic cells and monocytes, the latter of which can differentiate into pro-monocytes under the sustained action of M-CSF. Pro-monocytes can differentiate to form specifically marked monocytes, namely: LY6C^+^ or LY6C^-^ blood monocytes, under appropriate stimulation, such as M-CSF, TNF. Monocytes (Ly-6C^−^) induced by M-CSF develop into macrophages, but the addition of RANKL converts monocytes into osteoblasts commitment. Early-stage Ly-6C^+^ monocytes exhibit a high potential for osteoclast commitment in response to M-CSF and RANKL activation while still retaining the capacity to transform into Ly6C^-^monocytes. Completely differentiated macrophages induced by M-CSF and RANKL can fuse to become osteoclasts. Under pathological situations, macrophages can generate multinucleated giant cells (MGCs) when stimulated with M-CSF or stimulating factor (GM-CSF) and interleukins (IL-4, IL-13). MGCs continue to differentiate into osteoblasts in the presence of common fusion mediators. In addition, immature dendritic cells have the potential for osteolytic differentiation.

In the diverse colony-forming units (CFUs), which generate various myeloid cells, only the CFU macrophages, when exposed to M-CSF, evolve into macrophage and dendritic cell (DC) progenitor (MDP). MDP is a bipotent progenitor (24) which undergoes osteoclastic differentiation (25) (**Figure 1**). In addition to the differentiation of myeloid progenitors into osteoclasts, macrophages also serve as an osteoclastic source. Osteoclasts can be formed from tissue-specific macrophages in inflammatory and immunological environments (26) (**Figure 1**). For example, osteoclasts can be differentiated from osteal macrophages and synovial macrophages in an inflammatory condition (26, 27). Further, premature DCs can develop into typical DCs, while they can also become osteoclasts when exposed to M-CSF and RANKL (25, 28) (**Figure 1**).

On the basis of CD14 and CD16 antigens expression, monocytes are divided into three groups: classical (CD14^++^CD16^−^), intermediate (CD14^++^CD16^+^), and nonclassical (CD14^+^CD16^++^) (29, 30). According to this hypothesis, classical monocytes are the major source of osteoclasts, while intermediate monocytes can transform into high-absorption-capacity osteoclasts during inflammation, with non-classical monocytes evolving into non-resorbable osteoclasts (31–34). The study has reported that the intermediate monocytes convert into M1-macrophages to perform crucial functions in inflammation of the synovium. However, findings have shown that the classical monocytes account for the vast majority of monocytes in rheumatoid arthritis (RA) (35). This suggests that in an inflammatory environment, the main cells that are recruited are still classical monocytes, while the main role is played by M1-type macrophages which differentiate from intermediate monocytes. A recent study has identified an erythromyeloid progenitor (EMP)-derived osteoclast precursor population (36). Yolk-sac macrophages of EMP origin produced neonatal osteoclasts that can create a space for postnatal bone marrow hematopoiesis (36). Furthermore, EMPs gave rise to long-lasting OCPs that contribute to postnatal bone remodeling in both physiological and pathological settings (36). Single-cell RNA-sequencing data showed that EMP-derived OCPs arose independently of the HSC lineage, and the data from fate-tracking of EMP and HSC lineages indicated the possibility of cell-cell fusion between these two lineages (36). Cx3cr1^+^ yolk-sac macrophage descendants reside in the adult spleen, and parabiosis experiments showed that these cells migrated through the bloodstream to the remodeled bone after injury (36). Another study also found that the postnatal maintenance of osteoclasts, bone mass, and bone marrow cavity involves iterative fusion of circulating blood monocytic cells with long-lived osteoclast syncytia (37).

## Polarization of Macrophages: Influence on Osteoclasts

**Figure 2:**
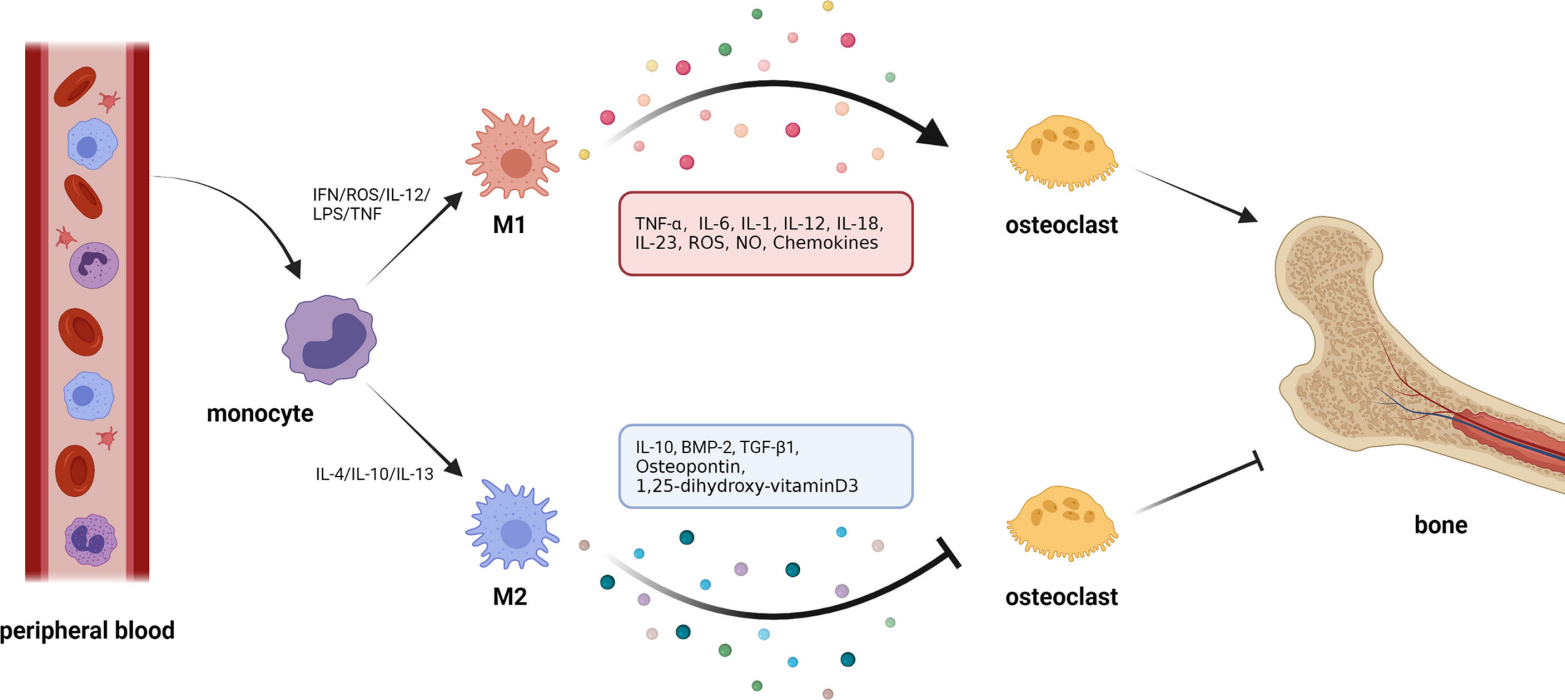
Macrophage polarization and osteoclasts. 1. Circulating macrophages change their phenotype according to the external environment. In the presence of cytokines, such as interferon (IFN), reactive oxygen species (ROS), interleukin-12 (IL-12), and tumor necrosis factor (TNF), macrophages polarize to M1 type. In response to interleukin-4 (IL-4), interleukin-10 (IL-10), interleukin-13 (IL-13), and other cytokines, macrophages can be polarized to M2 type. 2. Macrophages can produce cytokines with opposite effects in different polarization states. M1 macrophages can secrete cytokines, such as tumor necrosis factor-α (TNF-α), interleukin-6 (IL-6), interleukin-1 (IL-1), etc., which generally show the effect of activating osteoclasts and promoting bone resorption. M2 macrophages can secrete cytokines, such as IL-10, bone morphogenetic protein-2 (BMP-2), transforming growth factor-β1(TGF-β1), etc., most of which can inhibit osteoclastic bone resorption.

Since Elie Metchnikoff first defined these phagocytic cells over a century ago, the forms and roles of macrophages are becoming increasingly diverse (38). The ability of macrophages to modify their phenotypic features due to different external stimuli is known as activation (39, 40) (**Figure 2**). Alternatively activated macrophages (AAM; M2) were described in the early 1990s as possessing a distinct phenotype of macrophages from the traditionally activated or inflammatory macrophages (M1) (41). This categorization was developed as a result of the phenotypic changes observed *in vitro* after treatment with a variety of chemicals (42). Following the discovery of the M1/M2 macrophage paradigm, more evidence was found to support the idea that between these two seemingly opposed resultant phenotypes, there is a transitional form of intermediate phenotypes (39, 43, 44). Recently, a human macrophage with an open spectrum of activation, as identified by transcriptional clusters associated with diverse stimulations, was reported in a study. Under this condition, scholars have gradually coined the terminology ‘polarization’ to describe the disruption of macrophages by some stimuli, which induce various patterns of gene expression (40). However, the M1/M2 terminology was eventually accepted due to the discovery of different features of macrophages in cells generated from Th1 or Th2 dominant mouse lineages (42).

It is worth noting that the metabolic state of macrophages and osteoclasts is dependent on “activation” status, which itself is closely related to external stimuli or the disease environment. In pathological conditions, macrophages tend to be activated as M1-type macrophages that secrete proinflammatory cytokines against pathogens. Specifically, in inflammatory conditions, for example, following an infection, inflammatory stimuli, such as IL-12, lipopolysaccharide (LPS), interferon (IFN)-γ, and reactive oxygen species (ROS), that are generated by a damaged or necrotic tissue, promote polarization of macrophages into M1 phenotype (45–49). This is mediated by signal transducer and activator of transcription (STAT) 1, and interferon-regulatory factor (IRF) 5. As an important inflammatory response, polarized M1 can produce high levels of ROS, nitric oxide (NO), and proinflammatory cytokines, such as IL-1, IL-2, IL-6, IL-12, TNF-α, and IFN-γ, which are involved in enhancing the host’s defense response (50, 51). Under non-inflammatory conditions, macrophages largely exhibit the M2 phenotype which promotes tissue homeostasis and repair. Specifically, M2 macrophages are mainly present in the subsiding phase of inflammation, and they are responsible for the production of anti-inflammatory cytokines and the clearance of apoptotic cells. Exposure to anti-inflammatory cytokines (IL-4, IL-10, and IL-13), IL-1 receptor ligands, or immune complexes and Toll-like receptors (TLRs) can lead to M2 macrophage polarization *via* STAT6 and IRF4 (9, 52, 53). M2 macrophages can produce anti-inflammatory cytokines, such as chemokine (C-C motif) ligand 18 (CCL-18), CCL-22, IL-10, and a small amount of IL-12 family members (39, 54). In addition, M2 macrophages can produce a large number of osteogenic growth factors, such as bone morphogenetic protein-2 (BMP-2), a subclass of the TGF-β family and a potent promoter to osteogenic differentiation of MSCs (55, 56), TGF-β (57), osteopontin (58), and 1, 25-dihydroxyvitamin D_3_ (59). M1 macrophage-related cytokines, such as TNF-α, IL-6, and IL-1β, can induce osteoclastogenesis, while the M2 macrophage-related cytokines, such as IL-4 and IL-10, can inhibit osteoclastogenesis through the downregulation of NFATc1 (60, 61). Thus, the polarization of macrophages (M1/M2) itself is important for the determination of osteoclastogenesis, which makes the interaction between macrophages and osteoclasts even more complex.

Similar to macrophages, the metabolic state of osteoclasts is also closely related to the disease conditions. For instance, the seminal study of Trouillet-Assant et al. discovered that infection of bone marrow-derived OCPs with live *S. aureus* promoted their differentiation into activated macrophages rather than osteoclasts (62). However, the cultivation of OCPs with only the supernatants of the infected macrophages promoted the differentiation into bone-resorbing cells, due to the secreted proinflammatory cytokines they contained. Infection of mature osteoclasts with *S. aureus* promoted their bone-resorbing capacity in a hydroxyapatite matrix degradation assay which proceeded with enhanced cellular fusion events, and a higher number of nuclei per osteoclast with a significantly increased size (62). Similarly, when fractures occur, osteoclasts can be activated *via* the RANKL pathway (63). Subsequently, immature HSC precursors are recruited *via* CXCR4 and MMP-9 pathways to promote HSC migration (63).

### M1 Polarization and Osteoclasts

In inflammatory conditions, for instance, after an infection, inflammatory stimuli, such as IL-12, IFN-γ, and ROS, that are generated by damaged or necrotic tissue, promote the polarization of macrophages into M1 phenotype (45–49), resulting in the release of cytokines, such as TNF-α, IL-6, IL-1, and others to affect osteoclast differentiation and formation (**Table 1**).

**Table 1:** Summary of the effects of cytokines produced by M1 macrophage on osteoclasts.

Cytokine	Action	Reference
TNF-α	Promotes OC progenitors differentiation/amount; enhances RANKL secretion; facilitates the transformation from M2 to M1	(64–66)
IL-1α	Promotes RANKL and OC maker expression; stimulates MITF induction	(67, 68)
IL-1β	Promotes OC differentiation, survival; enhances RANKL secretion; employs negative feedback to attenuate osteoclast formation; exhibits time-dependent impacts on osteoclastogenesis; stimulates MITF induction	(67–72)
IL-6	Stimulates RANKL secretion; mediates the action of TNF-α, IL-1; activates JAK/STAT3 pathway	(73–75)
IL-12	Inhibits OC differentiation, activation, and survival; stimulates IFN-γ generation	(76–88)
IL-18	Synergizes with IL-12; stimulates IFN-γ generation; inhibits OC differentiation, formation, survival, activity, but induces osteoclastogenesis indirectly in RA	(84, 89–96)
IL-23	Promotes RANK and RANKL expression; induces osteoclastogenesis *via* regulating IL-17	(97–99)
CXCL2	Promotes OC progenitors proliferation	(100)
CXCL8	Up-regulates IL-6 synthesis	(101)
CXCL10	Increases RANKL and TNF-α expression	(102)
CXCL20	Upregulates IL-6 synthesis	(101)
CX3CL1	Increases OC adhesion to bone surface	(100)
CCL4	Increases OC migration	(103)
NO	Low levels of NO enhance osteoclastogenesis, whereas high levels suppress	(104–106)
ROS	Promotes OC differentiation; acts as a crucial second messenger during osteoclastogenesis; participates in the pathological process of osteoporosis	(107–112)

Osteoclastogenesis is promoted *via* TNF-α both directly by increasing OCP population and/or differentiation, and indirectly by enhancing RANKL secretion in osteoblasts and other cells (64, 65, 113). Moreover, TNF-α has been reported to be able to change CD11b^+^F4/80^+^ cells (bone marrow cells) from Ly6C^−^Gr1^−^M2 macrophages to Ly6C^−^Gr1^−^CD11c^+^ and Ly6C^+^Gr1^−^CD11c^+^ M1 macrophages. Pretreatment of M-CSF-stimulated mouse bone marrow with TNF-α increased osteoclast progenitor population, resulting in an increased number of osteoclasts generated by Ly6C-Gr1 and Ly6C^+^Gr1- monocyte series (66). This may indicate that the function of TNF-α is to boost osteoclastic precursors by changing the M-CSF-activated M2 differentiation to M1 macrophages, with elevated osteoclastogenesis potentials (66). IL-1 can also promote osteoclastogenesis by downregulating osteoprotegerin (OPG) levels and upregulating RANKL levels (113).

Lipocytes, muscle cells, and lymphocytes all generate IL-6, which is part of the cytokine family that also includes IL-11, IL-27, oncostatin M (OSM), and others (114). In response to injuries, IL-6 is generated locally and released into the bloodstream, where it triggers a rapid immune response (115). Osteocytes and osteoblasts have been shown to be potently stimulated by IL-6 to produce RANKL (73, 74). The interaction of IL-6/IL-6 receptor and glycoprotein 130 (gp130) receptor can ignite Janus-activated kinase (JAK) and cause the phosphorylation of STAT3, allowing RANKL to enhance osteoclastic development (74). Treatment with STAT3 blocker, CP690,550, decreased expression of the IL-6 family members, and also the generation of osteoclast both *in vivo* and *in vitro* (116). Importantly, the potential of IL-6 to stimulate osteoclastogenesis is determined by the presence of IL-6 receptors on osteoblasts, but not on OCPs (117). The membrane-bound IL-6 receptors (IL-6Rs), and the soluble IL-6 receptor (sIL-6R) are the two major transmitters that transduce IL-6 signals significantly. Interaction of IL-6 with IL-6R results in the dimerization with common signaling receptor subunit gp130, and subsequent downstream signaling transduction through phosphorylation of the JAK/STAT pathway (118). In cells without membrane-bound IL-6R, the interaction of IL-6 with sIL-6R results in the formation of a molecule that can combine with membrane-bound gp130 and activate downstream signaling (118). Soluble IL-6R signaling is hypothesized to be involved in the proinflammatory effects of IL-6 on several tissues. In addition, the effects of TNF-α and IL-1 on human osteoclastogenesis can also be mediated by IL-6 (75).

As a result of its significant bone-resorbing property, IL-1 was first classified as an osteoclast-activating factor (119). The adaptor molecule, MyD88, is activated by IL-1 to initiate signaling pathways that result in the initiation of NF-κB and MAPKs, as well as downstream transcription factors that promote gene expression and osteoclast formation (120). The IL-1 gene family contains a number of different members, including: IL-1α, IL-1β, and the IL-1R antagonist (IL-1Ra) (69). IL-1α and IL-1β are agonists, while IL-1Ra is a specialized receptor antagonist. Among these members, IL-1β is one of the key mediators of bone resorption in inflammatory conditions. IL-1β promotes TNF-α-stimulated osteoclast formation by improving the synthesis of RANKL in mesenchymal cells (70). In the presence of adequate RANKL concentrations, IL-1β directly promotes the development of OCPs under the regulation of p38 MAPK (70). Moreover, since IL-1R is localized on the osteoclast surface (71), IL-1β and other IL-1s can suppress the apoptosis of osteoclasts by activating the NF-κB pathway (121). However, IL-1β is a multifaceted cytokine with mostly pro-osteoclastogenesis features, but can also employ negative feedback mechanisms that attenuate osteoclast formation (69). IL-1β is greatly time-dependent in its impacts on osteoclastogenesis. As shown in earlier projects with mouse cells and as demonstrated in multiple experiments employing human OCPs (67, 72), exposure to IL-1β before or simultaneously with RANKL inhibits osteoclast formation. Conversely, exposure to IL-1β post-RANKL treatment has the reverse result, i.e., enhancing osteoclast development and resorptive activity. The reason for this phenomenon is that IL-1Rs share a cytosolic Toll-IL-1R domain and common intracellular signaling molecules with TLR, so IL-1β can directly block the initial phases of human osteoclast maturation by dropping of the M-CSF receptor, c-Fms, that is essential for RANK synthesis after binding with TLRs (72). In addition to IL-β, IL-1α is also known to be a potent osteoclastogenic cytokine. IL-1α also requires adequate RANKL concentrations to trigger the expression of OC biomarkers, such as tartrate-resistant acid phosphatase (TRAP), CtsK, MMP-9, and nuclear factor of activated T-cells cytoplasmic 1 (NFATc1) (68). Furthermore, regardless of RANKL, IL-1 may promote OC differentiation in bone marrow macrophages by activating the microphthalmia transcription factor (MITF) (67).

The cytokines, IL-12 and IL-23, which are reported to perform a key part in inflammatory responses, participate in the inflammation-induced bone abnormalities. IL-12, composed of p35 and p40, is one of the key factors influencing osteoclasts. IL-12 is mainly produced by macrophages and DCs, and is essential for Th cell formation and activity (76–78). IFN-γ is the main product of activated Th cells (79). This factor prevents osteogenic differentiation of bone marrow mesenchymal stem cells and causes damage to the cells (80). Notably, IFN-γ is also a powerful blocker of osteoclast differentiation. Briefly, it can inhibit osteoclast differentiation by decreasing CtsK levels (81) or increasing the decomposition of tumor necrosis factor receptor (TNFR)-associated factor 6 (TRAF6) (82, 83). In addition, IL-12 itself can also inhibit osteoclast activity (84–86). Mechanistically, IL-12 can downregulate NFATc1 expression, and thus inhibit RANKL-induced osteoclastogenic potentials (87). Also, IL-12 can promote TNF-α-induced osteoclast apoptosis *via* the FAS/FASL pathway (88). IL-23, composed of p40 and p19 subunits, is also a member of the IL-12 family. The overexpression of IL-23 has been reported to increase Th1 production and induce significant bone loss in mice (122). Further studies demonstrated that bone marrow macrophages isolated from IL-23 knockout mice possessed diminished osteoclast capacity and resorption efficiency (122). The two main mechanisms through which IL-23 positively regulates osteoclasts involve either directly increasing RANK expression in OCPs (97) or indirectly promoting RANKL production in CD4^+^ T cells (98). Furthermore, IL-23 can affect osteoclasts through its regulation of IL-17 secretion. In the presence of IL-17, the transformation of OCPs into osteoclasts is inhibited and the secretion of MMP-9 and CtsK by mature osteoclasts is reduced (99). Follow-up studies have shown that activation of NF-κB, p38, and ERK signaling pathways is the main mechanism by which IL-17 acts.

IL-18, like IL-12, also inhibits the formation of osteoclasts, and both have a synergistic effect (84). In combination with IL-12, IL-18 prevents the formation of osteoclasts more effectively (84). In addition, IL-18, similar to IL-12, was originally recognized as an IFN-inducing agent, and studies have demonstrated that the two cytokines work in tandem, with combination therapy leading to considerably more IFN-γ production than either IL alone (89–92). IFN-γ can induce cell apoptosis and suppress osteoclast differentiation. Of note, IL-18 can also inhibit osteoclast bone resorption partially mediated by IL-6 (93). Mechanistically, by modulating the synthesis of M-CSF and granulocyte-macrophage colony‐stimulating factor (GM-CSF) through T-cells, IL-18 inhibits the differentiation of osteoclasts, and induces apoptosis of osteoclasts by the generation of NO (94, 95). However, some studies have reported that in RA synovitis, IL-18 can indirectly induce osteoclastogenesis by upregulating RANKL synthesis through T cells (96). Therefore, the effects of macrophages on osteoclasts still need to be discussed.

Most commonly referenced ROS are hydroxyl radicals (HO·), hydrogen peroxide (H_2_O_2_), and hyperoxide (superoxide) anions (O^2-^) (123). It is important to note that cell reproduction, viability, apoptosis, maturation, motility, and metabolic activity are all regulated by ROS. ROS are critical secondary intracellular messengers involved in numerous physiological activities, such as apoptosis, epigenetics, and the initiation of cell signal transductions (124). ROS are primarily generated by mitochondrial metabolism, particularly by ETC complexes I and II, where oxygen utilization results in ROS generation due to the reverse electron transfer between the complexes and molecular oxygen. Also, ROS could be produced in the cytosol by the oxidative process of NADPH oxidases (NOX). It has been known for many years that the activities and development of osteoclasts are regulated by ROS (107, 108, 125–127). Exposure of macrophages to exogenous ROS, such as H_2_O_2_, causes the RANK signaling cascade to become activated, resulting in the development of osteoclasts (107), whereas RANKL initiation causes the synthesis of endogenous ROS, which subsequently functions as a second messenger to induce the conversion of macrophages into osteoclasts (109). RANKL can enhance the levels of intracellular ROS throughout osteoclastogenesis by initiating signaling pathways that include: TRAF6 and NOX1 (108). There is growing evidence that the activities of important osteoclast transcription factors, such as NF-κB and NFATc1, may also be influenced by ROS (110). Furthermore, the production of ROS by RANKL-stimulated osteoclasts has been shown to suppress the synthesis of antioxidant proteins like catalase (CAT) and superoxide dismutase (SOD) (111). As a result, ROS can be regarded as a crucial signaling messenger during osteoclastogenesis. In osteoclast-mediated diabetic osteoporosis, the over-activation of ROS/MAPKs/NF-κB/NLRP3 pathway is also the main cause of osteoclast resorption (112).

NO is a free radical that regulates many physiological activities, including vascular relaxation (128), neurotransmission, platelet aggregation, and immunological control (129). *In vivo*, NO is generated by the enzyme NO synthase (NOS) (130, 131). It is known as NOS for its ability to catalyze the oxidation of guanidine nitrogen from L-arginine in the presence of calcium ions, NADH, and tetrahydrobiopterin as co-factors (130, 131). NO and NOS have been shown to have a significant influence on bone cell activity in recent years. The effects of NO on skeletal structures have been shown to be extremely concentration-dependent, according to recent research reports (130, 131). The impacts of NO on osteoclast development and survival are similar to those seen in osteoblasts: a low level of NO enhances, whereas an excess of NO suppresses (104–106). *In vivo*, NOS1 performs necessary functions for the development and survival of osteoclasts because NOS1-lacking mice exhibited significantly reduced osteoclast production, and *in vitro*, NOS1-lacking bone marrow monocytes formed dysfunctional osteoclasts (106, 132). Also, the synthesis of NOS2 in OCPs is induced by RANKL, and NOS2 produces high NO concentrations that suppress osteoclastogenesis in a cGMP-independent manner (133). A subsequent study *in vitro* has shown that OCPs from NOS2-lacking mice were prone to differentiate more easily and form bone pits more quickly (133). Additionally, the production of NOS2 by IL-1 and IFN-γ has also been shown to inhibit the activities of osteoclasts (134–136).

Chemokines are dynamic molecules that are released in response to inflammatory conditions (137). They serve as a key regulator in osteoclast formation (138). Inflammatory osteo-disorders cause C-X-C motif chemokine ligand (CXCL)8, CXCL9, CXCL10, and chemoattractant chemokine ligand (CCL)20 concentrations to rise (101, 139). According to recent research reports, CXCL8 and CCL20 seem to have a contribution to osteoclast generation via regulating IL-6 synthesis in primordial osteoblasts (101). Likewise, CXCL10 enhances the secretion of RANKL and TNF-α in stimulated CD4^+^ T cells, which subsequently stimulates osteoclast formation (102). Mutually, CXCL10 synthesis in OC precursors is also induced by RANKL (102). CXCL2, which is produced by RANKL activation, promotes the procreation of OC progenitors by activating ERK (100). Osteoblast-derived CX3C chemokine ligand (CX3CL)1 enhances osteoclastogenesis by causing OC precursors to adhere to the site of bone resorption (100). CCL4 is a key regulator of OC motility through the promotion of PI3K activity, according to recent findings (103). In addition, CCL2 (MIP-1), CCL5 (RANTES), CCL7 (MCP-3), and CXCL12 (SDF-1) can also promote motility, adherence, absorption, and survivability of OC (140, 141).

### M2 Polarization and Osteoclasts

M2 macrophages are mostly found during the descending stages of infection and they secrete anti-inflammatory factors, e.g., IL-10 (39, 54). In addition, M2 macrophages can generate several osteogenic growth factors, such as BMP-2, TGF-β1 (57), OPN (58), and 1,25-dihydroxyvitamin D_3_ (1,25-(OH)_2_D_3_) (59). Through these factors, M2 macrophages further exert their influence on osteoblasts (**Table 2**).

**Table 2:** Summary of the effects of cytokines produced by M2 macrophage on osteoclasts.

Cytokine	Action	Reference
IL-10	Inhibits OC formation; reduces OC activity; upregulates OPG and downregulates RANKL, M-CSF; blocks the production of pro-osteoclast factors, like IL-1, IL-6, and TNF-α	(142–146)
BMP-2	Promotes OC formation, differentiation, activity, and survival; regulates RANKL and M-CSF production	(142–151)
TGF-β1	Inhibits NFATc1 expression and suppresses OC generation, and activity	(152)
OPN	Increases OC attachment; improves OC activity; regulates matrix calcification and cytokines, like IL-10, IL-12, IFN-γ	(153–157)
1,25-dihydroxy vitamin D_3_	Inconsistent effect on osteoclasts; inhibits OC formation and differentiation mostly, but promotes osteogenesis sometimes	(158–165)

As a founding member of the IL-10 cytokine family, IL-10 demonstrates immune suppressive properties in a wide spectrum. In addition to the IL-10 molecule, this cytokine community includes the IL-20 subfamily system (IL-19, IL-20, IL-22, IL-24) as well as the more genetically distinct IFN family (IL-28A, IL-28B, IL-29) (147, 148, 166). IL-10 is produced by a subpopulation of Th2 cells and possesses the ability to inhibit secretions by Th1 cells (149). Therefore, IL-10 was originally labeled as a secretory cytokine synthesis inhibitory factor (CSIF) (149). As research progresses, various immune cells, such as macrophages, DCs, mast cells, eosinophils, neutrophils, natural killer cells, CD4^+^ and CD8^+^ T cells, have been found to secrete IL-10. All of these cells are derived from the innate and acquired immune systems (150, 166). IL-10 has been demonstrated in some trials to prevent osteoclast formation (142, 143). Actually, through interference with NFATc1 activation and nuclear translocation, IL-10 is a robust osteoclastogenesis inhibitor (144). OPG synthesis is upregulated by IL-10, while RANKL and M-CSF expressions are downregulated (145). IL-10 suppresses osteoclastic activity by blocking the generation of pro-osteoclast factors, like IL-1, IL-6 and TNF-α (146). Therefore, IL-10 can be classified as an anti-osteoclastogenic cytokine.

BMP-2 is a member of the BMP family of proteins, and also belongs to the TGF cytokine superfamily (151). M2 macrophages are the main secretors of BMP-2 compared to M0- and M1-type macrophages. Previous studies have shown that BMP-2 becomes a potent inducer of bone remodeling by directly regulating osteoclast differentiation and osteoblast activity. Specifically, by regulating the synthesis of RANKL and M-CSF, BMP-2 organizes osteoclast differentiation and manages osteoclast survival, maturation, and activation (167–171). The significance of BMP-2-induced phosphatidylinositol 3-kinase and its downstream target AKT kinase has been identified in BMP-2-involved osteoblast maturation, and M-CSF secretion from osteoblasts to promote osteoclastogenesis (170, 172). In addition, through a self-regulatory circuit including Smad/Akt/Ca^2+^ signaling pathway, BMP-2 can also activate the NFATc1 transcription factor (173). NFATc1 promotes osteoclast differentiation through transcriptional activation of RANKL, which in turn stimulates the production of NFATc1 (174–176). It can be seen that BMP-2 performs a key function in the formation, development, and activation of osteoclasts.

TGF-β1, which is required for skeletal metabolism, can be synthesized by macrophages. The osteoclastogenic transcription factor, NF-κB, which is made up of proteins like p65, is part of the RANKL-RANK signaling pathway (177) and the main downstream controller of this pathway is NFATc1 (178). TGF-β1 has been demonstrated to limit the production and matrix degradation of osteoclast by directly inhibiting the expression of NFATc1 *via* obstruction p65 in the receptor activator (152).

OPN is a highly phosphorylated glycophosphoprotein with acidic properties and a high aspartic acid content (153). As a versatile factor, OPN is involved in the processes of inflammation, biomineralization, cell viability, and wound healing, as well as cardiovascular disease, cancer, diabetes, and renal calculus formation (153). OPN is secreted from osteoblasts and osteoclasts in osteoid (153). Research activities on mice with OPN genes knocked out show that the volume and length of the ruffled margins on the osteoclasts were several folds lower implying poorer resorptive potentials (154). In RA, OPN expressions can be aided by many factors in synovial tissues, and OPN is an active factor in the recruitment of osteoclasts (155) by acting as a coupling agent of osteoclasts to bones (156). Moreover, OPN affects organisms by modulating osteoclast activity and altering CD44 receptors, as well as through the secretion levels of cytokines, like IL-10, IL-12, IL–3, IFN-γ, integrin vB3, NF-κB (153). Furthermore, OPN, which is one of the most firmly coupled non-collagen proteins, is thought to aid in the adhesion of osteoclasts to the surface bones (156, 157). In summary, during the biomineralization stage, OPN has three key functions, namely: regulation of osseous cell attachment, regulation of osteoclastic activity, and regulation of matrix calcification.

The active form of vitamin D, i.e., 1,25-dihydroxyvitamin D_3_ (also called calcitriol), is a steroid molecule that balances calcium and bone metabolism, regulates cell proliferation and differentiation, and exerts immunoregulatory functions (179). Some research reports have shown that calcitriol possesses the ability to suppress osteoclastogenesis, while also promoting osteogenic differentiation (158, 159). In fact, 1,25-dihydroxyvitamin D_3_ can inhibit osteoclast differentiation by downregulating PAR2 mRNA expression which contributes to the determination of cells of osteoclast lineages (160). Furthermore, findings have uncovered the interactions between the BMP-Smad1 and IκB-NF-κB pathways in the process of osteoclast formation (161). Through the BMP-Smad1 and IκB-NF-κB pathways, 1,25-dihydroxyvitamin D_3_ can also influence osteoclast lineage commitment (161). Calcitriol suppresses osteoclastogenesis by altering the quantity and function of Th cell subgroups (Th2/Th17) under an inflammatory condition (162). However, there are also reports indicating the promoting role of 1,25-dihydroxyvitamin D_3_ in osteoclastogenesis. For example, applying high doses of calcitriol to congenital osteoporosis promotes the production of osteoclasts and generates certain effects (163). In addition, genetic effects of 1,25-dihydroxyvitamin D_3_ are exerted by its coupling of vitamin D receptor (VDR) to VDR response elements (VDREs) in the promoter sequences of target genes for vitamin D (164). More so, VDREs include genes of commonly-expressed proteins, such as RANKL, associated with adjustment of osteoclastogenesis (165). It can be seen that the role of 1,25-dihydroxyvitamin D_3_ in osteoclastogenesis needs to be further assessed.

## The Relationship Between Subgroups of Macrophages and Osteoclasts

Macrophages are a type of innate immune cells that may be present in almost every tissue and exert effects on immunological responses, wound-healing, and homeostasis (180). Macrophages that are distributed in various tissues are called tissue-resident macrophages. Tissue-resident macrophages were formerly thought of as differentiated monocytes that seeded the tissues and performed immunological sentinel and homeostatic activities as well as other tasks (181). However, these macrophages are not all the same, but rather a collection of cells that share a common set of functions and characteristics (181). In the next section, we will discuss the relationship between osteoclasts and subgroups of macrophages, mainly tissue macrophages.

### Osteal Macrophages and Osteoclasts

Osteal macrophages, a subgroup of bone-resident macrophages, are found right next to osteoblasts, where they govern bone production and play a variety of roles in the homeostasis of the skeleton (182). It is known that osteal macrophages can support the function of osteoblasts and promote bone anabolism, but the relationship between osteal macrophages and osteoclasts is still unclear. Myeloid progenitor cells give rise to osteoclasts and osteal macrophages (183), yet research findings have revealed that osteoclast formation is the more robust or preferable access for myeloid lineage differentiation (184). Although osteal macrophages can become osteoclast under the stimulation of RANKL and M-CSF, monocytes and other myeloid progenitors have been discovered to be more effective OCPs (185). In addition, osteoclasts and osteal macrophages possess unique membrane characteristics that distinguish them from one another. Osteal macrophages are independent of osteoclasts, having F4/80 positivity but TRAP negativity (185). As reported in recent studies, osteal macrophages were shown to exhibit both typical membrane antigens of macrophage-like CD68 and more specialized antigens, like Mac-3 and CD169 (182, 186, 187). These findings showed that osteal macrophages are a malleable, yet distinctive cell type with specialized roles in the microenvironment of the bone marrow. However, osteal macrophages can indeed influence osteoclasts. A recent study found that osteal macrophages perform a unique part in promoting osteoclast activity, as well as the first proof of their participation in the pathogenesis of osteoporosis (188). Additionally, osteal macrophages are involved in the effect of PTH on osteoclasts (184). When stimulated by local stimuli, osteal macrophages tend to release pro- and anti-osteoclastogenic factors, such as TNF-α (65, 189), IL-6 (190), IL-1 (189, 191), or IFN-β (192, 193), regulating osteoclast production and activity. In general, the relationship between osteal macrophages and osteoclasts needs further exploration.

### Tumor-Associated Macrophages and Osteoclasts

The term “tumor-associated macrophages” (TAMs) refers to macrophages that have been attracted from circulating monocytes to tumors and have been impacted by the presence of cancer to facilitate tumor aggressiveness and development (194). TAMs are made up of M2 cells and a few M1 cells (195). Research has proven that the recruited monocytes in tumor sites are capable of being differentiated into TAMs and osteoclasts (196). Although TAMs and osteoclasts have a similar precursor, however, bisphosphonates have been demonstrated to precisely target TAMs (197, 198). Notably, TAMs are also a source of osteoclasts. According to previous studies, when exposed to cancer cells, 1,25-(OH)_2_D_3_, and M-CSF or to M-CSF and RANKL, TAMs have been reported to transform into functional bone-resorbing osteoclasts (18, 199). Moreover, under different polarization states, TAMs can release numerous cytokines, like IL-10 (200), IL-12 (201), which regulate osteoclast generation and function. In some tumor metastasis, TAMs and osteoclasts have similar roles (202, 203). Overall, the mechanisms regarding the interaction between TAMs and osteoclasts remain unclear and this area needs to be further explored. CD4^-^ macrophages have a positive effect on osteoclast differentiation.

### Synovial Macrophages and Osteoclasts

Specialized lining cells of the synovial intima, as well as macrophages found in the synovial subintima and synovial fluid, represent synovial macrophages (204). Similar to monocyte macrophages in other organs, they are produced from a common bone marrow progenitor and show some functional and biological properties (205). The most distinguishing characteristic of these cells is their proclivity for phagocytosis. However, they also perform a variety of other functions, including the initiation and modulation of hormones and cellular immunity, as well as the generation and discharge of a great number of secretory substances (205). A study has proven that synovial macrophages can develop into mature osteoclasts with lacunar resorption capabilities, but this process requires TNF-α/IL-1 or M-CSF along with RANKL (27). Because inflammatory synovial fluids have an increased number of macrophages and higher concentrations of RANKL, TNF-α, and IL-1, synovial macrophages in rheumatoid or crystal arthritis have a stronger potential to generate osteoclasts. Furthermore, a study has shown that OCPs in the synovium are monocytes/macrophages that express the CD14 antigen (206). In RA and inflammatory OA, CD14^-^ macrophages have a positive effect on osteoclast differentiation of CD14^+^ macrophages (206). Overall, the exploration of the relationship between synovial macrophages and osteoclasts will help researchers better understand the therapy and pathogenesis of diseases, such as OA and RA.

### Other Macrophage Subgroups and Osteoclasts

In addition to traditionally activated M1 macrophages, researchers recently found that human atherosclerotic plaques contain macrophages that express the mannose receptor (MR), an alternative macrophage identifier, implying that plaque macrophage populations are heterogeneous (207). This specific type of M2 macrophage, being positive for both CD68 and MRs, can develop into osteoclast-like cells (OLCs) and have a poor display of RANKL-stimulated osteoclastic bone resorption (208, 209). Osteoclasts and microglia are separate tissue-resident macrophages found in the bone and brain where they cause pathological alterations of osteoporosis and Alzheimer’s disease (AD), respectively (210). Both of them share critical signaling pathways, including three receptor signaling pathways, namely: TREM2/DAP12, M-CSF, and CCR5, which converge to control actin-microtubule dynamics and cytoskeleton architecture *via* the Pyk2 signaling pathway (210). Research has shown that AD patients are more prone to develop osteoporosis than the general population (210). Alveolar macrophages and osteoclasts have certain similarities, and therefore bisphosphonates can inhibit both cell types. A study has further shown that alendronate suppresses macrophage migratory and phagocytotic functions, as well as the inflammatory sensitivity of alveolar macrophages by blocking NF-κB signaling pathway (211). Albeit, alendronate inhalation ameliorates elastase-induced pulmonary emphysema by inducing apoptosis of alveolar macrophages (211). In addition, a case has been reported in which alveolar macrophages can be transformed into osteoclasts (212). In addition to the above cells, epithelioid and foam cells also exhibit certain associations with osteoclasts (213, 214).

## Conclusion and Future Perspectives

Macrophages and osteoclasts have always been a hot and difficult area of research in osteoimmunity. Previous studies tended to focus on a certain type of cell and seldom summarize the latest updates on the interconnection between these two myeloid origin cells for the field of osteoimmunology. However, in this paper, we have outlined present knowledge on the relationships between macrophages and osteoclasts, including their common origin, regulation during polarization, and subgroups’ interactions. Be that as it may, the study of these two cellular associations still has many aspects that need to be further explored, and perplexing difficulties still remain. Notably, current studies tend to focus on the unidirectional effect of macrophages on osteoclasts during polarization at the expense of the effect of osteoclasts on macrophages. Actually, osteoclasts can be involved in the production of various cytokines, which act on macrophages. Here, we hypothesize that there are interactions among macrophages, osteoclasts, and osteoblasts to maintain bone homeostasis. Therefore, we believe that the imbalance between these cells in disease states and its specific mechanisms will be a new field for further exploration. Additionally, as a member of the mononuclear macrophage system (215), osteoclasts are considered as the resident macrophages in bone (182) and play a phagocytic role similar to that of macrophages. The similarities and differences between the two types of cells in performing phagocytosis are also an aspect worth investigating. A recent study reveals that besides apoptosis, RANKL-stimulated osteoclasts have an alternative cell fate in which they undergo fission into daughter cells called osteomorphs, once resorption is complete (216). Osteoclasts recycle *via* osteomorphs during RANKL-stimulated bone resorption (216). As an unexplored therapeutic target, the interaction of osteomorphs with macrophages will also be a new area of research.

The ultimate objective of practically all biomedical exploration is to improve patient outcomes and develop novel medicines, and renewed efforts in osteoimmunity will be important in accomplishing this translational goal. Currently, most therapies for bone-related diseases primarily focus on one specific cell type, but lack definite approaches aiming at the connections among cells in the bone microenvironment. Thus, the pharmaceutical targets in terminating the pathological association of macrophages with osteoclasts in disease states could become a noteworthy concern in the nearest future.

## Author Contributions

YS drafted the manuscript. JL and XX assisted in reviewing literature. FG, ZS, and KZ modified the manuscript. YS and TY reviewed and edited the final manuscript. TY revised the manuscript. All authors contributed to the article and approved the submitted version.

## Funding

This article was supported by the National Natural Science Foundation of China (Grant No. 31970090).

## Conflict of Interest

The authors declare that the research was conducted in the absence of any commercial or financial relationships that could be construed as a potential conflict of interest.

## Publisher’s Note

All claims expressed in this article are solely those of the authors and do not necessarily represent those of their affiliated organizations, or those of the publisher, the editors and the reviewers. Any product that may be evaluated in this article, or claim that may be made by its manufacturer, is not guaranteed or endorsed by the publisher.
